# Supplementary material for: ProteinShader: illustrative rendering of macromolecules
Source: BMC Struct Biol. 2009 Mar 30;9:19. doi: 10.1186/1472-6807-9-19 (PMC2672931; doi:10.1186/1472-6807-9-19)
Supplement: Additional file 1 — ProteinShader program without source code. This compressed file contains the complete ProteinShader program including associated libraries, but no source code. A README.txt file gives an overview of the ProteinShader distribution, and the index.html file in the help subdirectory has directions on getting started with the program as well as a set of tutorials. [file 1472-6807-9-19-S1.zip › ProteinShader-beta-0_9_4-binary/help/api/org/proteinshader/graphics/textures/EqualSignConfigReader.html]

EqualSignConfigReader (ProteinShader API)


|  |  |  |
| --- | --- | --- |
| **Overview** \| **Package** \| **Class** \| **Use** \| **Tree** \| **Deprecated** \| **Index** \| **Help** | |  |
| **PREV CLASS**   **NEXT CLASS** | **FRAMES**    **NO FRAMES**     **All Classes** |  |
| SUMMARY: NESTED \| FIELD \| CONSTR \| METHOD | DETAIL: FIELD \| CONSTR \| METHOD |  |


---


## org.proteinshader.graphics.textures Class EqualSignConfigReader

```
java.lang.Object
  org.proteinshader.graphics.textures.EqualSignConfigReader
```

**All Implemented Interfaces:**: ConfigReader

---

``` public class EqualSignConfigReader extends Object implements ConfigReader ```

Reads a configuration file to obtain a list of menu names
and matching filenames for textures.

---

**Constructor Summary**

`EqualSignConfigReader()`
  
          Constructs an EqualSignConfigReader object.


| **Method Summary** | |
| --- | --- |
| `List<NamePair>` | `readConfigFile(File file)`             Reads a configuration file to obtain a list of menu names with a matching filename for each menu name. |
| `List<NamePair>` | `readConfigFile(String filename)`             Reads a configuration file to obtain a list of menu names with a matching filename for each menu name. |

**Methods inherited from class java.lang.Object**

`clone, equals, finalize, getClass, hashCode, notify, notifyAll, toString, wait, wait, wait`

**Constructor Detail**

### EqualSignConfigReader

```
public EqualSignConfigReader()
```

:   Constructs an EqualSignConfigReader object.


**Method Detail**

### readConfigFile

```
public List<NamePair> readConfigFile(String filename)
                              throws TextureException
```

:   Reads a configuration file to obtain a list of menu names with a matching
    filename for each menu name.

---


### readConfigFile

```
public List<NamePair> readConfigFile(File file)
                              throws TextureException
```

:   Reads a configuration file to obtain a list of menu names with a matching
    filename for each menu name.
      
      
    The file is expected to be an equal sign separated file of the form   
    menu name 1 = filename 1   
    menu name 2 = filename 2   
    menu name 3 = filename 3   
    etc.

    :   **Specified by:**: `readConfigFile` in interface `ConfigReader`
    :   **Parameters:**: `file` - the config file to read. **Returns:**: A list of menu name and filename pairs. **Throws:**: `TextureException` - if the config file cannot be read.


---


|  |  |  |
| --- | --- | --- |
| **Overview** \| **Package** \| **Class** \| **Use** \| **Tree** \| **Deprecated** \| **Index** \| **Help** | |  |
| **PREV CLASS**   **NEXT CLASS** | **FRAMES**    **NO FRAMES**     **All Classes** |  |
| SUMMARY: NESTED \| FIELD \| CONSTR \| METHOD | DETAIL: FIELD \| CONSTR \| METHOD |  |


---

# *Copyright © 2007-2008*
